# Childhood maltreatment and intimate partner violence in dissociative disorder patients

**DOI:** 10.3402/ejpt.v5.24568

**Published:** 2014-09-12

**Authors:** Aliya R. Webermann, Bethany L. Brand, Gregory S. Chasson

**Affiliations:** Department of Psychology, Towson University, Towson, MD, USA

**Keywords:** Dissociation, dissociative identity disorder, dissociative disorders, intimate partner violence, domestic violence, childhood maltreatment, childhood abuse

## Abstract

**Background:**

Childhood maltreatment (CM) is a risk factor for subsequent intimate partner violence (IPV) in adulthood, with high rates of retrospectively reported CM among IPV victims and perpetrators. A theorized mechanism of the link between CM and IPV is dissociation. Dissociation may allow perpetrators of violence to remain emotionally distant from their behavior and minimize empathy toward those they victimize, enabling them to commit acts of violence similar to their own experiences. Indeed, elevated rates of dissociation and dissociative disorders (DD) have been found among IPV survivors and perpetrators. In addition, in pilot studies, DD clinicians have reported high levels of violent behavior among DD patients.

**Objective:**

The present study investigates IPV among DD patients with Dissociative Identity Disorder and Dissociative Disorder Not Otherwise Specified, a group with CM rates of 80–95% and severe dissociative symptoms.

**Methods:**

DD clinicians reported on rates of CM and IPV among 275 DD patients in outpatient treatment. DD patients also completed a self-report measure of dissociation. Analyses assessed the associations between CM typologies and IPV, as well as trait dissociation and IPV.

**Results:**

Physical and emotional child abuse were associated with physical IPV, and childhood witnessing of domestic violence (DV) and childhood neglect were associated with emotional IPV.

**Conclusions:**

The present study is the first to provide empirical support for a possible CM to adult IPV developmental trajectory among DD patients. Future research is needed to better understand the link between CM and IPV among those with trauma and DD.

Intimate partner violence (IPV), which encompasses physical, sexual, psychological, or emotional harm from a current or former partner, is a frequent and harmful occurrence (Centers for Disease Control and Prevention, 2013). Within the United States, in their lifetime, one in four women and one in seven men endure severe physical abuse, one in five women and 1 in 71 men experience rape, and about half of men and women experience psychological aggression (Centers for Disease Control and Prevention, 2011). The consequences of IPV include a multitude of medical health issues, including asthma, diabetes, and irritable bowel syndrome, as well as a range of mental health issues, such as posttraumatic stress disorder, major depressive disorder, and generalized anxiety disorder (Centers for Disease Control and Prevention, 2011; Felitti et al., 1998). The societal impact of IPV is especially significant for women; 81% of women and 35% of men who experience IPV report an adverse health or safety outcome, such as emergency medical treatment of injuries, shelter stays, and feeling fearful for one's life (Centers for Disease Control and Prevention, 2011; Melton & Belknap, 2003).

Male and female survivors of sexual violence report predominately male perpetrators, while for emotional and physical abuse, men report predominately female perpetrators and women report predominately male perpetrators (Centers for Disease Control and Prevention, 2011). IPV perpetrators tend to commit multiple offenses against their partners (Rand & Saltzman, 2003) and go on to victimize numerous individuals (Lisak & Miller, 2002). Thus, exploring mechanisms of abuse could facilitate the development of prevention programs that reduce interpersonal violence.

A promising approach to deterring adult IPV may involve prevention of, or intervention in, childhood maltreatment (CM). CM has been proposed as a causal factor in IPV perpetration due to the high rates of CM reported by interpersonally violent offenders. This includes domestic violence (DV) offenders in a battering intervention program (Dutton, 1995; Simoneti, Scott, & Murphy, 2000); sex offenders in prison (Hulnick, 1997) and treatment (Becker-Blease & Freyd, 2007; Ellason & Ross, 1999); male undergraduates (Lisak, Hopper, & Song, 1996); incarcerated violent offenders (Swica, Lewis, & Lewis, 1996); and incarcerated homicide defendants (Lewis, Yeager, Swica, Pincus, & Lewis, 1997). While most CM survivors do not become IPV perpetrators (Lisak et al., 1996), it is feasible that much IPV is perpetrated by CM survivors.

One theorized factor that may link CM and IPV is dissociation, a particularly insidious and destructive psychological consequence of CM. The myriad negative medical and mental health outcomes of CM have been well-documented in retrospective (Felitti et al., 1998; Putnam, Harris, & Putnam, 2013) and longitudinal studies (e.g., Trickett, Noll, & Putnam, 2011). Dissociation is a potential response to severe early childhood trauma, especially ongoing physical and sexual abuse, as it allows children to protect themselves from the full traumatic impact (Loewenstein & Putnam, 1990; Putnam, 1997), compartmentalize their experiences (Ludwig, 1983; van der Hart, Nijenhuis, & Steele, 2006), cope with fear and dependence on an abusive caregiver (Freyd, De-Prince, & Zurbriggen, 2001), and increase pain tolerance (Giolas & Sanders, 1992; Nijenhuis, Vanderlinden, & Spinhoven, 1998; Schauer & Elbert, 2010). Severe dissociative symptoms that continue beyond their adaptive capacity may lead to dissociative identity disorder (DID), which involves a compartmentalization of the individual's identity into self-states (distinct and alternate personality states and identities), accompanied by amnesia (American Psychiatric Association, 2013). The etiology of DID and Dissociative Disorder Not Otherwise Specified (DDNOS) in CM is supported through such patients evidencing CM rates from 80 to 95% (e.g., Boon & Draijer, 1993; Brand et al., 2009; Dalenberg et al., 2012; Ellason, Ross, & Fuchs, 1999; Putnam, Guroff, Silberman, Barban, & Post, 1986; Ross & Ness, 2010; Saxe, Chinman, Berkowitz, & Hall, 1994; Saxe, van der Kolk, Berkowitz, & Chinman, 1993; Yargiç, Şar, Tutkun, & Alyanak, 1998).

Dissociation has been posited to play a causal role in violence perpetration. It may allow a perpetrator to emotionally distance from the offense and maintain minimal empathy for their victim, especially for survivors of violence who perpetrate acts similar to abuse they experienced (Egeland & Susman-Stillman, 1996; Moskowitz, 2004; Ross, 2008). Research on interpersonally violent offenders has demonstrated elevated trait dissociation, increased rates of DD, and higher retrospectively reported peritraumatic dissociation (i.e., acute dissociative responses as traumatic events unfold; Marmar, Metzler, & Otte, 2004) for their own CM experiences and while perpetrating abuse toward others (Becker-Blease & Freyd, 2007; Ellason & Ross, 1999; Hulnick, 1997; Leibowitz, 2007; Lewis et al., 1997; Ross, 2008; Simoneti et al., 2000; Swica et al., 1996).

The connection between dissociation and violence has also been examined through preliminary studies on clinician-reported violence perpetration among DD patients. Significant correlations between patients’ trait dissociation and sexual aggression have been found among psychiatric inpatients (Quimby & Putnam, 1991). In addition, several studies have reported high amounts of interpersonal violence perpetration, especially among male patients: dissociated self-states that exhibit internal (i.e., within the patients’ mind) or external (i.e., acted out toward others) violent behavior in 70–90%, violent criminal convictions in 10–29%, homicide in 6–19%, rape in 13–20%, and incarceration in 10–29% (Loewenstein & Putnam, 1990; Putnam et al., 1986; Ross & Norton, 1989). One study to date, using a subset of the same sample as the present study (derived from Brand et al., 2013) examined DD patients' revictimization and perpetration toward others in 6-month increments over the span of 30 months. Revictimization rates were high, ranging from 3.5 to 36%, and a quarter of those who were victimized also perpetrated abuse toward others (Myrick, Brand, & Putnam, 2013). However, in contrast to the present study, the Myrick et al. (2013) study utilized a small sample of longitudinal data and does not link IPV to types of CM.

As DD patients report staggering rates of severe and frequent CM, exhibit clinically significant dissociation, and have a high prevalence of violent self-states and behaviors, it follows that they would be at elevated risk for involvement in an abusive intimate relationship. Currently, there are few published articles connecting DD to IPV, and to our knowledge, only one on IPV in DD patients (Myrick et al., 2013). The majority of studies focus on children's exposure to familial DV as a causal factor in dissociation. There are articles discussing dissociation as a predictor of revictimization (e.g., Iverson et al., 2013; Noll, Horowitz, Bonanno, Trickett, & Putnam, 2003), reports of elevated dissociation among abused women (e.g., Marchiori, Rossi, & Colombo, 2004; Stein, 2012), as well as articles demonstrating dissociation as a risk factor for sexually transmitted infections among women with CM and IPV histories (e.g., Sutherland, 2011; Sutherland, Fantasia, & Adkison, 2014).

Thus, the present study aims to address the gap in research on CM, dissociation, and IPV. A start in this inquiry is to examine IPV among DD patients, as well as evaluate statistical associations between CM and IPV in this clinical population. We evaluated the association between physical and emotional IPV and five types of CM: physical, emotional, and sexual abuse; childhood witnessing of DV; and neglect. We also investigated the link between IPV and trait dissociation. We hypothesized that all types of CM and trait dissociation would be significantly associated with physical and emotional IPV. This is the first study to provide empirical support for a possible CM to IPV developmental trajectory among DD patients.

## Methods

### Participants

The present study utilized data from the longitudinal, naturalistic, and prospective Treatment of Patients with Dissociative Disorders (TOP DD) study (Brand et al., 2009, 2013). Further information on the study's methodology can be found in Brand et al. (2009). DD clinicians were recruited to participate in the study and volunteered one of their current patients diagnosed with DID or DDNOS. Data were collected over 30 months. The current study used only Time 1 data because attrition rates were nearly 50% for both patients and clinicians (Brand et al., 2013) over the course of the TOP DD study, and the current investigation of CM and IPV was not longitudinal in scope. The present study utilized data on 275 patients. Demographics are as follows: 94% female, 5% male, and 1% transgender; 89% Caucasian, 2% African American, 2% Hispanic, 2% Asian, and 5% other; age *M*=43.7, *SD*=10.7, range=18–72. This sample's demographics approximate the typical clinical sample of Caucasian females with DD (Brand et al., 2009).

### Clinician measures

#### Intimate partner violence

Physical IPV was assessed through clinicians responding to the question: “Has the patient been in a physically abusive relationship in the past 6 months?” Emotional IPV was assessed through clinicians responding to the question: “Has the patient been in an emotionally abusive relationship in the past 6 months?” If the clinician responded yes to either question, they were asked whether the patient was the victim, perpetrator, or both. Clinicians were not asked about other types of IPV (e.g., sexual IPV).

#### Childhood maltreatment

CM was assessed by asking clinicians if their patients had experienced the following forms of CM: physical abuse, emotional/psychological abuse, sexual abuse, childhood witnessing of DV, and neglect. Clinicians responded yes, unclear, or no.

### Patient measures

#### Trait dissociation

Trait dissociation was measured with the Dissociative Experiences Scale-II (DES-II; Carlson & Putnam, 1993). DES-II is a widely used 28-item measure in which the participant indicates what percentage of the time a particular dissociative experience occurred within the past month. An example item is: *Some people find that sometimes they are listening to someone talk and they suddenly realize that they did not hear part or all of what was said*. A meta-analysis of the DES-II by van Ijzendoorn and Schuengel (1996) demonstrated test–retest reliability of *r*=0.78–0.93, internal consistency of *α*=0.93, and convergent validity of *r=*0.67. Internal consistency for the current sample was *α*=0.95.

### Analyses

Chi-square tests were conducted to assess the association between CM and IPV, given that both variables were categorical. Two-tailed independent samples *t*-tests were conducted to assess the association between IPV and trait dissociation, the latter of which is a continuous variable. In addition, *Z*-tests were conducted to compare IPV rates in the present study versus the general population, using US national IPV statistics. Inflation of experiment-wise Type I error rate was addressed through the False Discovery Rate (FDR) control procedure for determining statistical significance when conducting multiple hypothesis tests (Benjamini & Hochberg, 1995). FDR decreases Type I error rates while maintaining statistical power, making it preferable over more conservative corrections (Verhoeven, Simonsen, & McIntyre, 2005). Based on this procedure, *α*_critical_=0.014. All analyses were included in the FDR correction. As an effect size, odds ratios (ORs) were calculated to predict the likelihood of a participant being in an abusive adult relationship if they experienced a particular type of CM.

## Results

Clinicians reported high rates of IPV in their patient participants. All those who perpetrated IPV also experienced IPV victimization (henceforth referred to as *victim-perpetrators*), precluding chi-square analysis that specifically compares victim-perpetrators to those who perpetrated IPV but did not experience IPV victimization. As a result, the victim-perpetrator subcategory was collapsed into the victim subcategory. Thus, an affirmative IPV status in the present study should be conceptualized as involvement in an abusive relationship, rather than parsing out those who have been victimized, those who have perpetrated, or both.

### Prevalence of childhood and adult abuse

According to clinician report, 29.6% of patients had experienced physical IPV within the last 6 months; 26.1% of the sample was physically victimized, and 3.5% of the sample was a victim-perpetrator of physical IPV. In addition, 58.8% of patients experienced emotional IPV within the last 6 months; 48.9% of the sample was emotionally victimized, and 9.8% of the sample were victim-perpetrators of emotional IPV.

As aforementioned, clinicians were asked about patients’ history of five types of CM and responded by answering yes (coded as 1), unclear (coded as 2), or no (coded as 3). Clinicians’ response codes were summed into a total composite score of CM history. The score had a possible range of 5–15, with high scores indicating less CM. A score of 5 indicates a yes response on all types of CM, whereas a score of 15 indicates a no response on all types of CM. Patients averaged a high rate of CM (*M=*6.7, *SD*=1.7, Median=6), and the range was 5–13, meaning that every patient was at least suspected to have experienced CM. CM rated as *unclear* by clinicians were removed from analyses in order to increase the interpretability of the results (*N*s removed: physical abuse=44, DV=85, neglect=60, sexual abuse=33). The exception was child emotional abuse, as there were only yes and unclear responses, which necessitated keeping the unclear cases to compare to the definite emotional abuse cases (*N* unclear=20).

### IPV in sample versus population

*Z*-tests were conducted to compare the proportion of IPV in the present study's sample of DD patients versus IPV in the general population. Population IPV rates were obtained from the Centers for Disease Control and Prevention's National Intimate Partner and Sexual Violence Survey (2011). The proportion of physical IPV in the present study's sample, 29.6%, was not significantly different from the general population, 25%, *Z*=1.66, *p=*0.089. However, the proportion of emotional IPV in the present study's sample, 58.8%, was significantly different from the general population, 48.8%, *Z*=3.33, *p*<0.014.

### Physical IPV

Significant associations were found between physical and emotional child abuse and physical IPV (Table 1). There was a significant association between child physical abuse and physical IPV, *χ* ^2^(1)=9.60, *p*<0.014. The odds of physical IPV were 12.80 (95% CI: 1.66–98.64) times higher for those who experienced child physical abuse compared to those who did not. There was also a significant association between experiencing child emotional abuse and physical IPV, *χ* ^2^(1)=11.41, *p*<0.014. The odds of physical IPV were 14.65 (95% CI: 1.93–111.08) times higher for those who experienced child emotional abuse compared to those for whom their child emotional abuse history was unclear.

**Table 1:** Childhood maltreatment and intimate partner violence

CM	*χ* ^2^	Frequencies of IPV vs. no IPV	% Likelihood of IPV vs. no IPV	ORs (95% CI)	*χ* ^2^	Frequencies of IPV vs. no IPV	% Likelihood of IPV vs. no IPV	ORs (95% CI)
		Physical IPV				Emotional IPV		
Physical abuse	9.60^a^	99/1	46.0/6.3	12.80 (1.66–98.64)		170/9	79.1/56.3	N/A
Emotional abuse	11.41^a^	111/1	43.5/5	14.65 (1.93–111.08)		200/11	78.4/55	2.82 (1.47–5.80)
Witnessing DV	2.85	87/8	47.3/25.8	N/A	9.82^a^	108/34	81.2/59.6	2.97 (1.33–6.61)
Neglect	4.96	57/17	42.9/29.8	N/A	7.55^a^	148/18	80.4/58.1	N/A
Sexual abuse	0.020	98/4	42.1/44.4	N/A		178/7	76.4/77.8	N/A

IPV =intimate partner violence; CM=childhood maltreatment; OR=odds ratio; CI=confidence interval; N/A=odds ratios were not calculated as chi-square tests were non-significant; DV=domestic violence.

Some chi-square tests could not be conducted because the minimum cell count was not met.

DF=1 for all *χ* ^2^ tests.

a *p*<0.014, based on FDR *α*_critical_.

Significant associations were not found between physical IPV and either childhood witnessing of DV [*χ* ^2^(1)=2.85, *p*=0.091], exposure to childhood neglect [*χ* ^2^(1)=4.96, *p*=0.026], or sexual CM [*χ* ^2^(1)=0.020, *p*=0.887]. There was also not a significant difference in DES-II scores among those who experienced physical IPV (*M=*36.42, *SD*=19.19) compared to those who did not (*M*=35.86, *SD*=20.30), *t*(212)=0.204, *p*=0.840. This was also found when the sample was split into those meeting the DD cutoff score (i.e., DES-II scores ≥30; Moskowitz, 2004), *t*(119)=−1.037, *p=*0.302 and those not meeting the DD cutoff score (i.e., DES-II scores <30), *t*(91)=−1.257, *p*=0.212.

### Emotional IPV

Significant associations were found between childhood witnessing of DV and childhood neglect and emotional IPV (Table 1). There was a significant association between exposure to DV and emotional IPV, *χ* ^2^(1)=9.82, *p*<0.014. The odds of emotional IPV were 2.92 [95% CI: 1.47–5.80] times higher for those exposed to DV compared to those who were not. There was also significant association between childhood neglect and emotional IPV, *χ* ^2^(1)=7.55, *p*<0.014. The odds of emotional IPV were 2.97 [95% CI: 1.33–6.61] times higher for those who experienced childhood neglect compared to those who did not.

Chi-square analysis could not be conducted for the association between emotional IPV and child physical, emotional, or sexual abuse because the cells did not have a minimum expected count of 5. There was not a significant difference in DES-II scores among those who experienced emotional IPV (*M=*36.00, *SD*=19.93) versus those who did not experience emotional IPV (*M=*35.96, *SD*=19.64), *t*(213)=0.010, *p*=0.992. This was also found when the sample was split into those meeting the DD cutoff score (i.e., DES-II scores ≥30; Moskowitz, 2004), *t*(120)=0.556, *p=*0.579, and those not meeting the DD cutoff score (i.e., DES-II scores<30), *t*(92)=−0.776, *p*=0.440.

## Discussion

The present study assessed the association between CM and recent IPV, as well as the association between trait dissociation and recent IPV. Our hypothesis that all CM typologies and trait dissociation would be significantly associated with physical and emotional IPV was partially supported. Significant associations were found between child physical and emotional abuse and physical IPV, with CM survivors evidencing high odds of experiencing physical IPV. For emotional IPV, significant associations were found with childhood witnessing of DV and childhood neglect, although the upper and lower limits of odds of emotional IPV were much narrower and generally lower compared to physical IPV. The likely explanation for the lower ORs for emotional IPV was that the sample evidenced a higher rate of emotional IPV (58.8%) than physical IPV (29.6%), so the sample was more likely to experience emotional IPV no matter their CM history. Trait dissociation (DES-II) was not significantly associated with IPV. This was found when using the entire sample, as well as when the sample was split into only those with DES-II scores meeting the DD cutoff (i.e., DES-II scores ≥30) and only those with DES-II scores not meeting the DD cutoff (i.e., DES-II scores<30; Moskowitz, 2004). It is possible that dissociation is not a mediator between CM and IPV. Future research should investigate this possibility further and include participants with a wide range of dissociative symptoms.

Several conclusions can be drawn from these findings. First, our sample of DD patients reported higher rates of emotional IPV victimization (58.8%) than the general public (48.8%; Centers for Disease Control and Prevention, 2011). The same conclusion cannot be drawn with perpetration, as reliable US national statistics on perpetration are difficult to obtain. This finding fits with past literature demonstrating high rates of IPV victimization and perpetration among CM survivors, as well as literature on elevated trait and peritraumatic dissociation among IPV victims and perpetrators. Second, certain types of CM were found to be significantly associated with physical and emotional IPV among DD patients. The relationship between CM and IPV victimization and perpetration has been found in other research, but the present study is the first to statistically demonstrate this relationship among DD patients.

The findings of the present study are relevant for clinicians working with DD and other psychiatric patients. As IPV is common among DD patients, all DD patients should be screened for IPV victimization and perpetration. All clinicians treating CM survivors, whether they are DD patients or not, should take an active role in helping to prevent IPV perpetration and revictimization among their patients. In addition, when working with DD patients and CM survivors, safety should be prioritized given their high prevalence of IPV. Clinicians should be knowledgeable about IPV referrals (e.g., hotlines and shelters), as well as evidence-based treatment for IPV victimization and perpetration.

The study is not without limitations. First, the sample is homogenous in gender, racial, and ethnic identity. The typical profile of a DD patient presenting for treatment is a Caucasian female (Brand et al., 2009), which presents difficulties for obtaining a diverse clinical sample. Thus, the effects found in the current study may not generalize to all individuals with DD, although the findings can speak to the typical DD patient in treatment. Second, our assessment of patient IPV and CM was limited by utilizing only clinician reports, and we did not assess all types of IPV (e.g., sexual IPV). We did not include patient self-reports on IPV or CM nor a validated assessment of CM or IPV. Patients may be reticent to report IPV and CM due to social stigma, shame, or guilt, so utilizing patient self-reports could increase the accuracy in reporting, and a validated measure could increase confidence when drawing conclusions about IPV and CM in this population. Indeed, future research using anonymous self-report measures may find that IPV perpetration and victimization rates are more frequent than previously documented, given that individuals with DD evidence two risk factors for IPV (i.e., CM history and severe dissociation). Third, we were required to merge IPV victims and IPV victim-perpetrators together into the same group due to the universality of IPV victimization among perpetrators. As a result, we could not parse out group differences between perpetrators who were also victims versus perpetrators who were not victims, nor differences in their IPV experiences based on trauma history, gender, or culture. Fourth, all research assessing IPV among DD patients, including the aforementioned preliminary studies, have limitations related to accuracy of IPV reports: DD patients may inaccurately perceive internal events among dissociated self-states as external violent events, leading them to report violent behavior toward others that did not occur (Putnam et al., 1986); or patients may confuse childhood events with present day events (International Society for the Study of Trauma and Dissociation, 2011).

As discussed, the current findings could be enhanced in future research by including patient self-reported data on IPV victimization and perpetration. Self-reported data on IPV, obtained through IPV measures such as the Revised Conflict Tactics Scale (CTS2; Straus, Hamby, Boney-McCoy, & Sugarman, 1996), Dissociative Violence Questionnaire (Simoneti et al., 2000), or through clinician and researcher interviews, has been successfully obtained in samples of highly dissociative violent offenders (Becker-Blease & Freyd, 2007; Dutton, 1995; Leibowitz, 2007; Lewis et al., 1997; Simoneti et al., 2000; Swica et al., 1996). A well-validated and short IPV measure, such as the Revised Conflict Tactics Scale-Short Form (CTS2S; Straus & Douglas, 2004) could be given as an optional measure. To avoid causing considerable distress during assessment, such measures could include a disclaimer that it references adult trauma and be given only to patients currently in treatment so they have supports in place.

In summary, the present study adds to the extant literature on the association between CM and adult IPV, and is the first to demonstrate this association in DD patients. The findings of this study give credence to the necessity of clinicians regularly screening for all types of IPV, especially among those with a history of CM, such as DD patients. Researchers should continue to examine the potential developmental trajectory from CM to IPV.
